# Clinical, Hematologic, and Inflammatory Factors Associated with Early Mortality and Postoperative ICU Admission After Geriatric Hip Fracture Surgery

**DOI:** 10.3390/life16101696

**Published:** 2026-10-10

**Authors:** Emre Tekşan, Selami Kaya

**Affiliations:** Department of Orthopaedics and Traumatology, Giresun University Faculty of Medicine, Giresun Training and Research Hospital, Giresun 28200, Türkiye

**Keywords:** hip fracture, geriatric, mortality, intensive care, C-reactive protein, red blood cell distribution width, blood transfusion

## Abstract

**Background**: This study evaluated whether routinely available admission hematologic and inflammatory markers remained associated with postoperative intensive care unit (ICU) admission and 1-month mortality when considered alongside established clinical and perioperative factors. **Methods**: We retrospectively analyzed 598 patients aged 65 years or older who underwent proximal femoral nailing (PFN) or hemiarthroplasty for hip fracture between October 2021 and October 2025. Admission laboratory values were obtained from the first blood tests at presentation. The primary outcome was 1-month mortality, and the secondary outcome was postoperative ICU admission. Group comparisons used the Mann–Whitney U and chi-square tests, and multivariable logistic regression was used for adjusted analyses. Given the limited number of deaths, the mortality analysis was considered exploratory. **Results**: Among 594 patients in the clinical ICU comparison, 228 (38.4%) were admitted to the ICU. One-month mortality occurred in 41 of 596 patients (6.9%). ICU admission was associated with age (OR 1.04, 95% CI 1.01–1.06), ASA score (OR 2.04, 95% CI 1.45–2.87), and number of packed red blood cell (PRBC) units transfused (OR 1.60, 95% CI 1.36–1.88). Mortality was associated with age (OR 1.07, 95% CI 1.02–1.12), male sex (OR 3.30, 95% CI 1.66–6.56), the fracture-treatment pathway represented by hemiarthroplasty versus PFN (OR 3.69, 95% CI 1.78–7.64), CRP per 10 mg/L (OR 1.05, 95% CI 1.01–1.10), and greater PRBC exposure (OR 1.37 per unit, 95% CI 1.11–1.70). RDW-CV was not associated with either outcome after adjustment. **Conclusions**: The observed associations with procedure and transfusion should not be interpreted as causal effects. Early outcomes were associated with multiple baseline, inflammatory, and perioperative factors, and the mortality findings remain exploratory and require confirmation in prospectively characterized cohorts.

## 1. Introduction

Geriatric hip fractures are a major public health problem as populations age. They are followed by substantial functional decline and mortality, with 1-year mortality commonly reported in the 20–30% range [1].

Mortality after hip fracture is multifactorial and is influenced by both patient-related and perioperative factors. Advanced age, male sex, higher American Society of Anesthesiologists (ASA) grade, comorbidity burden, and frailty are repeatedly reported as adverse prognostic factors [2]. Reduced physiological reserve and frailty may contribute to the increased vulnerability of older patients after fracture and surgery.

Several routinely available hematologic and biochemical markers have been studied for prognostic value after hip fracture. Red blood cell distribution width (RDW), a measure of variability in erythrocyte size, has been linked to systemic inflammation and oxidative stress and has been associated with mortality in systematic reviews and meta-analyses [3].

Inflammatory markers have also been examined. CRP-based indices, particularly the C-reactive protein-to-albumin ratio, have been associated with early mortality in geriatric hip fracture patients [4]. Higher inflammatory activity may reflect the combined effects of trauma, comorbidity, and perioperative stress.

Perioperative blood loss and transfusion are also relevant to early outcomes. Blood transfusion has been associated with mortality after hip fracture surgery [5], although transfusion requirement may reflect blood loss, anemia, and overall clinical severity.

These observations have encouraged multivariable assessment of demographic, clinical, and laboratory factors rather than reliance on a single marker [6].

Although these factors have been investigated extensively, uncertainty remains regarding their relative associations when routinely available clinical, inflammatory, and perioperative variables are considered simultaneously, particularly across both early mortality and postoperative ICU use. Treatment-related exposures such as surgical procedure and transfusion are also strongly influenced by indication and perioperative severity, which complicates causal interpretation in retrospective cohorts.

Accordingly, this study aimed to evaluate whether routinely available admission hematologic and inflammatory markers remained associated with postoperative ICU admission and 1-month mortality when considered alongside established clinical and perioperative factors, and whether the pattern of associations differed between the two outcomes.

## 2. Materials and Methods

### 2.1. Study Design and Patient Selection

This retrospective cohort study was conducted in the Department of Orthopaedics and Traumatology at Giresun Training and Research Hospital. Patients aged 65 years or older who underwent surgical treatment for hip fracture with proximal femoral nailing (PFN) or hemiarthroplasty between October 2021 and October 2025 were eligible for inclusion. Exclusion criteria were age younger than 65 years, nonoperative management, pathological fracture, periprosthetic fracture, multiple trauma, and records lacking data required for the relevant analysis. Active infection and chronic inflammatory disorders were not specific exclusion criteria.

**Figure 1:**
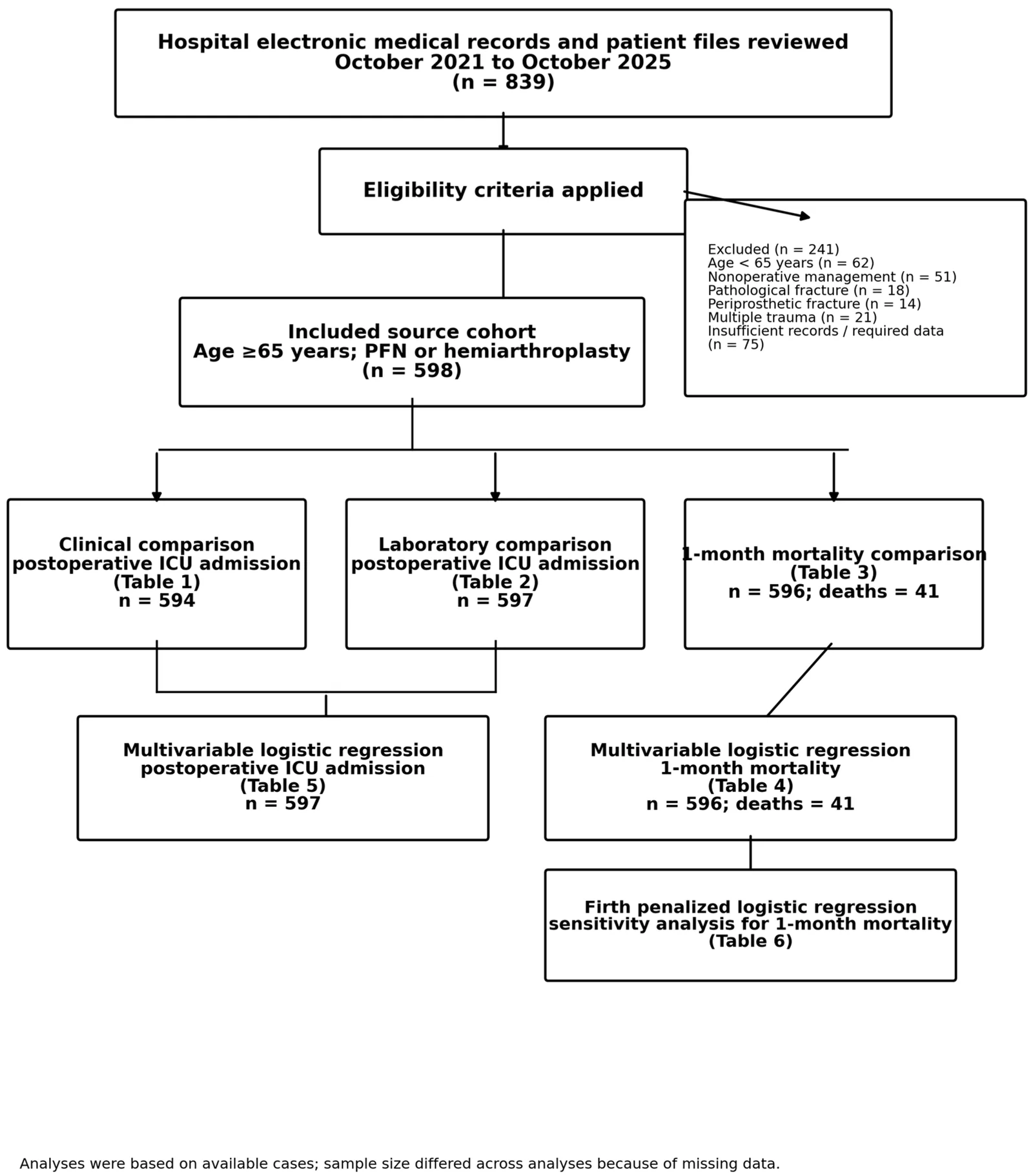
Patient flow diagram showing selection of the study cohort and the number of patients included in each analysis. The boxes identify the patient cohorts used for the analyses in Tables 1–6; arrows indicate the flow from screening to the analysis cohorts.

Hospital electronic medical records and patient files were reviewed retrospectively. A total of 839 patient records were assessed for eligibility. After application of the predefined inclusion and exclusion criteria, 241 patients were excluded, and the source cohort comprised 598 patients (Figure 1). Analyses were based on available cases for each comparison; because missingness differed across variables and outcomes, the analytic sample size ranged from 594 to 597. The denominator used for each analysis is reported in the corresponding table or results paragraph. Variable-specific missingness consisted of age in 1 patient, BMI in 3 patients, and WBC in 1 patient. Pre-fracture Parker Mobility Score was available for all 598 patients. One-month mortality status could be reliably confirmed for 596 of 598 patients using the hospital information system and the national Death Notification System; 2 patients could not be included in the mortality analyses because 1-month outcome status could not be reliably confirmed. Analyses were performed using available complete cases, resulting in analytic denominators of 594–597.

### 2.2. Ethical Approval

The study protocol was approved by the Clinical Research Ethics Committee of Giresun Training and Research Hospital (Decision No. 05, dated 5 April 2026). The study was conducted in accordance with the principles of the Declaration of Helsinki.

### 2.3. Data Collection

Demographic and clinical data were recorded from hospital records. Admission laboratory values were defined as the first blood test results obtained at initial hospital presentation before surgery. Pre-fracture mobility was recorded using the Parker Mobility Score (range 0–9), with higher scores indicating greater pre-fracture mobility. Parker Mobility Score was available for all 598 patients. The variables evaluated included:

- Age;
- Sex;
- Body mass index (BMI);
- American Society of Anesthesiologists (ASA) score;
- Surgical type (proximal femoral nailing [PFN] or hemiarthroplasty);
- Anesthesia type (spinal or general);
- Pre-fracture Parker Mobility Score;
- Admission hemoglobin (Hb, g/dL);
- Postoperative hemoglobin (day 0 and day 1, g/dL);
- Delta hemoglobin (admission–postoperative day 1, g/dL);
- Admission red blood cell distribution width (RDW-CV, %);
- Admission C-reactive protein (CRP, mg/L);
- Admission white blood cell count (WBC, ×10^3^/μL);
- Number of packed red blood cell (PRBC) units transfused.

Fracture location and surgical procedure were structurally linked in this cohort: intracapsular femoral neck fractures were treated with hemiarthroplasty, whereas extracapsular intertrochanteric fractures were treated with PFN. Consequently, fracture category and surgical procedure were perfectly collinear.

### 2.4. Outcome Measures

The primary outcome was 1-month mortality. Mortality status at 1 month after surgery was assessed using both the hospital information system and the national Death Notification System, allowing deaths occurring after hospital discharge to be captured. One-month mortality status was reliably confirmed for 596 of 598 patients; for 2 patients, 1-month outcome status could not be reliably confirmed, and these patients were excluded from mortality analyses. The secondary outcome was postoperative intensive care unit (ICU) admission. No single mandatory institutional threshold or scoring system was used for postoperative ICU admission. Planned ICU need could be documented during the preoperative anesthesia assessment on the basis of age, ASA physical status, major cardiopulmonary comorbidity, and overall perioperative risk. Unplanned ICU admission was determined intraoperatively or during emergence when clinically significant deterioration occurred, including major blood loss, delayed emergence, persistent hemodynamic instability, need for vasoactive support, respiratory insufficiency, or need for advanced respiratory support or close monitoring. Final disposition was determined by the attending anesthesiologist. For the mortality analysis, the transfusion variable represented the cumulative number of PRBC units administered from hospital admission through discharge, including preoperative, intraoperative, and postoperative transfusions. For the ICU analysis, only PRBC units administered before ICU admission were included. PRBC transfusion decisions were individualized and based on hemoglobin concentration together with the patient’s clinical condition, hemodynamic status, ongoing blood loss, symptoms or signs of anemia, and relevant cardiopulmonary comorbidities. No single mandatory hemoglobin threshold was applied to all patients.

### 2.5. Statistical Analysis

Continuous variables were presented as median (interquartile range [IQR]), and categorical variables were expressed as number (percentage).

For between-group comparisons, the Mann–Whitney U test was used for continuous variables and the chi-square test for categorical variables.

Multivariable logistic regression analyses were used to assess factors associated with postoperative ICU admission and 1-month mortality. The same core covariates were entered in both models: age, ASA score, sex, surgical type, admission hemoglobin level, RDW-CV, CRP level, and number of PRBC units transfused. A common core covariate set was maintained across both outcomes to preserve analytical consistency and avoid outcome-specific variable selection. The core covariates represented the principal clinical, hematologic, inflammatory, and perioperative factors relevant to the study objectives and were not selected solely according to statistical significance in unadjusted comparisons. BMI and anesthesia type were considered secondary clinical variables and were therefore evaluated in the unadjusted analyses but were not included in the primary multivariable models. Avoiding additional covariates also limited model complexity, which was particularly important for the mortality analysis given the small number of deaths. The Parker Mobility Score was evaluated in the unadjusted ICU and mortality comparisons. Because fracture category and surgical procedure were perfectly collinear, they were not entered simultaneously in multivariable models. Missing data were handled using complete-case analysis, and no imputation was performed. Multicollinearity was assessed using variance inflation factors (VIFs). Linearity in the logit for continuous predictors (age, admission hemoglobin, RDW-CV, CRP, and PRBC units) was assessed by comparing the linear specification with a cubic spline specification. Where nonlinearity was detected in the ICU model, age and CRP were modeled flexibly using cubic splines in a sensitivity analysis. Results were reported as odds ratios (ORs) with 95% confidence intervals (CIs); for continuous predictors, ORs correspond to the unit increment specified in the corresponding table. Because the mortality analysis included only 41 deaths, that model was considered exploratory. As a sensitivity analysis for the limited number of mortality events, Firth-penalized logistic regression was performed using the same core covariates, followed by a second Firth model additionally including the Parker Mobility Score. For the conventional logistic regression models, model discrimination was summarized using the area under the receiver operating characteristic curve (AUC), and internal calibration was summarized using calibration intercept and slope after bootstrap resampling (1000 resamples).

Primary analyses and conventional logistic regression were performed using IBM SPSS Statistics for Windows, version 26.0 (IBM Corp., Armonk, NY, USA). Firth penalized logistic regression sensitivity analyses were conducted in R version 4.3.2 (R Foundation for Statistical Computing, Vienna, Austria) using the logistf package (version 1.26.1). Additional VIF, spline-based, and model-performance diagnostic analyses were performed as supplementary model diagnostics.

## 3. Results

Demographic and clinical characteristics according to postoperative ICU admission are presented in Table 1. This comparison included 594 patients, of whom 228 (38.4%) were admitted to the ICU. Patients admitted to the ICU were older (*p* < 0.001), had a lower BMI (*p* = 0.001), and had higher ASA scores (*p* < 0.001). Surgical type (*p* = 0.019) and anesthesia type (*p* = 0.045) also differed between groups, whereas sex distribution did not (*p* = 0.531). Although the median Parker Mobility Score was 6.0 in both groups, the score distributions differed significantly (ICU: 6.0 [IQR 4.0–8.0] vs. non-ICU: 6.0 [4.0–9.0]; *p* = 0.018). BMI and anesthesia type were not included in the adjusted analyses.

Laboratory parameters according to postoperative ICU admission are presented in Table 2. Admission hemoglobin and postoperative hemoglobin levels on days 0 and 1 were lower in the ICU group (all *p* < 0.001). RDW-CV (*p* = 0.004), CRP (*p* < 0.001), and the number of PRBC units transfused (*p* < 0.001) were higher in the ICU group. Delta hemoglobin (*p* = 0.169) and WBC (*p* = 0.645) did not differ significantly between groups.

Demographic, clinical, and laboratory characteristics according to 1-month mortality are presented in Table 3. One-month mortality status was reliably confirmed for 596 patients, of whom 41 (6.9%) died within 1 month. Non-survivors were older (*p* < 0.001), were more frequently male (*p* = 0.004), and differed in surgical type (*p* < 0.001). ASA score was also higher among non-survivors (*p* = 0.044). Pre-fracture Parker Mobility Score did not differ significantly between survivors and non-survivors (6.0 [4.0–9.0] vs. 5.0 [4.0–7.0], respectively; *p* = 0.269). CRP (*p* = 0.004) and the number of PRBC units transfused (*p* = 0.003) were higher in non-survivors. Admission hemoglobin (*p* = 0.391), RDW-CV (*p* = 0.756), and WBC (*p* = 0.251) were not significantly different.

Table 4 presents the multivariable logistic regression analysis for 1-month mortality. After adjustment, age (OR = 1.07; 95% CI 1.02–1.12; *p* = 0.005), male sex (OR = 3.30; 95% CI 1.66–6.56; *p* < 0.001), the fracture-treatment pathway represented by hemiarthroplasty compared with PFN (OR = 3.69; 95% CI 1.78–7.64; *p* < 0.001), CRP (OR = 1.05 per 10 mg/L; 95% CI 1.01–1.10; *p* = 0.030), and greater PRBC exposure (OR = 1.37 per unit; 95% CI 1.11–1.70; *p* = 0.004) remained associated with mortality. ASA score, admission hemoglobin, and RDW-CV were not significant in the adjusted model. Firth penalized logistic regression produced materially similar estimates. After additional adjustment for the Parker Mobility Score, age, male sex, the fracture-treatment pathway, CRP, and PRBC exposure remained associated with mortality, whereas the Parker Mobility Score itself was not independently associated with 1-month mortality (OR 1.00, 95% CI 0.87–1.14; *p* = 0.956). Given the limited number of mortality events, this multivariable mortality model should be interpreted as exploratory.

Table 5 presents the multivariable logistic regression analysis for postoperative ICU admission. In the adjusted model, age (OR = 1.04; 95% CI 1.01–1.06; *p* < 0.001), ASA score (OR = 2.04; 95% CI 1.45–2.87; *p* < 0.001), and number of PRBC units transfused (OR = 1.60; 95% CI 1.36–1.88; *p* < 0.001) were associated with ICU admission. Male sex, surgical type, admission hemoglobin, RDW-CV, and CRP were not significant. BMI and anesthesia type were not included in the regression model.

VIF values for the core covariates ranged from 1.02 to 1.45, indicating no relevant multicollinearity. Spline-based assessment showed no evidence of nonlinearity for any continuous predictor in the mortality model (all *p* ≥ 0.204). In the ICU model, evidence of nonlinearity was observed for age (*p* = 0.016) and CRP (*p* = 0.003), whereas admission hemoglobin, RDW-CV, and PRBC units showed no evidence of nonlinearity (all *p* ≥ 0.152). When age and CRP were modeled using cubic splines, ASA score (OR = 1.98; 95% CI 1.40–2.79; *p* < 0.001) and PRBC units transfused (OR = 1.62; 95% CI 1.38–1.91; *p* < 0.001) remained associated with ICU admission. Because CRP showed evidence of nonlinearity in the ICU model, the single linear CRP OR reported in Table 5 should be interpreted cautiously.

The Firth penalized logistic regression sensitivity analyses for 1-month mortality are presented in Table 6. For the conventional logistic regression models, discrimination and internal calibration were summarized without a separate table. The mortality model had an AUC of 0.82, a calibration intercept of −0.03, and a calibration slope of 0.84; corresponding values for the ICU model were 0.76, 0.01, and 0.96, respectively.

## 4. Discussion

In this cohort, the findings can be considered across three clinical domains: baseline patient vulnerability, admission inflammatory/hematologic markers, and perioperative or treatment-related factors. Age and greater PRBC exposure were associated with both postoperative ICU admission and 1-month mortality; ASA score was associated with ICU admission, whereas male sex, the fracture-treatment pathway represented by hemiarthroplasty, and CRP were associated with mortality. RDW-CV did not retain an adjusted association, and pre-fracture Parker Mobility Score was associated with ICU admission only in the unadjusted comparison and was not independently associated with mortality in the Parker-adjusted Firth model.

Among baseline patient factors, older age was associated with both outcomes, consistent with a recent systematic review and meta-analysis identifying age as a predictor supported by high-quality evidence for 30-day mortality after hip fracture surgery [7]. Male sex was associated with 1-month mortality, in line with previous population-based and meta-analytic findings [7,8]. ASA score was associated with postoperative ICU admission but not with 1-month mortality; the prior literature supports its relationship with perioperative risk and early mortality [7,9]. Together, these findings suggest that baseline vulnerability contributes to early postoperative outcomes, although the absence of an ASA-mortality association in our model may partly reflect covariate overlap and the limited number of deaths.

The distribution of pre-fracture Parker Mobility Scores differed between ICU groups on unadjusted comparison despite identical median values of 6. In contrast, Parker Mobility Score did not differ significantly according to 1-month mortality and was not independently associated with mortality after inclusion in the Firth sensitivity model. These findings suggest that pre-fracture mobility may capture part of the baseline vulnerability relevant to postoperative monitoring needs, while it did not materially alter the observed mortality associations in this cohort.

Among admission biomarkers, CRP was associated with 1-month mortality, corresponding to an OR of 1.05 per 10 mg/L increase. Previous studies have reported similar associations between CRP-based inflammatory indices and early mortality after hip fracture [4,10]. However, CRP is nonspecific and may reflect the inflammatory response to fracture trauma, occult or clinically apparent infection, chronic inflammatory disease, or overall illness severity. Active infection and chronic inflammatory disorders were not specifically excluded in this cohort, which limits causal or disease-specific interpretation of the CRP association. RDW-CV, in contrast, was higher in ICU patients only on unadjusted analysis and was not associated with either outcome after adjustment. Although prior studies and meta-analyses have linked RDW to mortality after hip fracture [3,11,12], RDW-CV did not retain an independent association after adjustment in this cohort.

Hemiarthroplasty was associated with higher 1-month mortality than PFN. A large nationwide cohort from Türkiye also reported an association between surgical procedure and mortality after hip fracture [13]. In the present cohort, surgical procedure was structurally determined by fracture location: intracapsular femoral neck fractures were treated with hemiarthroplasty, whereas extracapsular intertrochanteric fractures were treated with PFN. Fracture category and procedure were therefore perfectly collinear and could not be entered simultaneously in the same multivariable model. Accordingly, the observed procedure-related association should be interpreted as reflecting the combined fracture-treatment pathway rather than an isolated effect of hemiarthroplasty itself. The association remained similar after adjustment for pre-fracture Parker Mobility Score in the Firth sensitivity analysis. Anesthesia type also differed between ICU groups in the unadjusted comparison. In routine practice, anesthetic technique was selected according to the patient’s clinical condition, comorbidity profile, and perioperative anesthetic assessment, so this unadjusted difference may partly reflect clinical selection. Because anesthesia type was not included in the adjusted model, the present data do not support an independent effect of anesthetic technique on ICU admission.

Because age, ASA status, and overall perioperative risk were themselves considered when determining postoperative ICU disposition, the observed associations with ICU admission are susceptible to incorporation or selection bias. Accordingly, ICU admission in this study should be interpreted primarily as reflecting postoperative monitoring and resource-utilization requirements rather than physiological deterioration alone.

Greater PRBC exposure was associated with both ICU admission and mortality, consistent with previous hip fracture cohorts [5,14]. For the ICU analysis, only transfusions administered before ICU admission were counted, whereas the mortality analysis included cumulative preoperative, intraoperative, and postoperative PRBC exposure from admission through discharge. Transfusion should therefore be interpreted as a perioperative treatment exposure that may also reflect blood loss, anemia, surgical complexity, and overall clinical severity rather than as an isolated causal determinant of adverse outcomes. Because the mortality analysis used cumulative PRBC exposure through discharge, some postoperative transfusions may have occurred after clinical deterioration. PRBC should therefore be interpreted as a perioperative exposure rather than a baseline prognostic factor, and reverse causation cannot be excluded.

The Nottingham Hip Fracture Score illustrates why early outcomes after hip fracture are considered in the context of multiple clinical variables rather than a single laboratory marker [15]. In our cohort, the pattern of associations likewise involved baseline, inflammatory, and perioperative factors, while RDW alone did not retain an adjusted association.

### Limitations

This study has several limitations. The retrospective single-center design limits generalizability and leaves room for residual confounding. Frailty and other established determinants of early mortality, including comorbidity burden, cognitive impairment, renal or cardiovascular disease, and timing of surgery, were not systematically captured in the analytic dataset in a sufficiently complete manner for multivariable adjustment. Frailty has consistently been associated with postoperative mortality and other adverse outcomes after hip fracture surgery in systematic reviews and meta-analyses [16,17]. Pre-fracture mobility was assessed using the Parker Mobility Score; however, this measure does not capture all dimensions of frailty or functional reserve. Fracture category was available but was perfectly collinear with surgical procedure because all intracapsular femoral neck fractures were treated with hemiarthroplasty and all extracapsular intertrochanteric fractures were treated with PFN. Therefore, the independent contributions of fracture category and procedure to mortality could not be separated statistically. The two fracture-treatment pathways also differ in surgical exposure and perioperative characteristics, which may contribute to residual confounding. BMI and anesthesia type were evaluated only in unadjusted analyses. Postoperative ICU disposition was based on attending-anesthesiologist judgment without a single mandatory institutional threshold, so this outcome may reflect local practice and resource use in addition to patient physiology. A reliable numerical breakdown of planned versus unplanned ICU admissions could not be reconstructed consistently from the retrospective records. Because age, ASA status, and overall perioperative risk were considered in ICU disposition decisions, associations with ICU admission may also be affected by incorporation or selection bias and should be interpreted partly as reflecting monitoring and resource-utilization practices. One-month mortality was assessed using both the hospital information system and the national Death Notification System; however, status could not be reliably confirmed for 2 patients. Inflammatory markers were measured only at admission, and active infection or chronic inflammatory disorders were not specifically excluded; consequently, CRP may reflect several sources of systemic inflammation. Missing data caused small differences in denominators across analyses. PRBC exposure in the mortality analysis was cumulative from admission through discharge and should be regarded as a perioperative treatment exposure that may also reflect blood loss, anemia, surgical complexity, or overall illness severity; the observational design cannot isolate a causal effect of transfusion itself. Because the mortality analysis used cumulative PRBC exposure through discharge, some postoperative transfusions may have followed clinical deterioration, and reverse causation cannot be excluded. Directly quantified perioperative blood loss was not available for multivariable adjustment. Finally, the mortality model included eight covariates but only 41 deaths, increasing the risk of overfitting; Firth penalized regression was therefore used as a sensitivity analysis, and the main associations were materially preserved. The mortality calibration slope of 0.84 was consistent with some degree of optimism, reinforcing the exploratory interpretation of the mortality model.

## 5. Conclusions

Among older patients undergoing PFN or hemiarthroplasty for hip fracture, age and greater PRBC exposure were associated with both postoperative ICU admission and 1-month mortality. Male sex, the fracture-treatment pathway represented by hemiarthroplasty, and CRP were associated with mortality, whereas RDW-CV did not retain an association after adjustment. Pre-fracture Parker Mobility Score was associated with ICU admission only in the unadjusted comparison and was not independently associated with mortality in the Firth sensitivity analysis. Because fracture category and procedure were perfectly collinear, the procedure-related mortality difference should be interpreted within the combined fracture-treatment pathway rather than as an isolated procedural effect. These findings suggest that early postoperative outcomes were associated with multiple baseline, inflammatory, and perioperative factors rather than with RDW alone. The mortality findings remain exploratory and require confirmation in prospectively characterized cohorts with standardized outcome definitions and more complete measures of frailty and functional status.

## Figures and Tables

**Table 1:** Demographic and Clinical Characteristics According to Postoperative ICU Admission.

Variable	Total (*n* = 594)	No ICU (*n* = 366)	ICU (*n* = 228)	*p*-Value
Age, years	84.0 (76.0–90.0)	82.0 (74.0–89.0)	87.0 (81.0–91.0)	**<0.001**
Body mass index (BMI), kg/m^2^	27.0 (24.0–29.0)	27.0 (25.0–29.0)	26.0 (24.0–29.0)	**0.001**
Sex, *n* (%)				
Female	414 (69.7)	259 (70.8)	155 (68.0)	0.531
Male	180 (30.3)	107 (29.2)	73 (32.0)	
Surgical type, *n* (%)				
PFN	342 (57.6)	225 (61.5)	117 (51.3)	**0.019**
Hemiarthroplasty	252 (42.4)	141 (38.5)	111 (48.7)	
Anesthesia type, *n* (%)				
Spinal	503 (84.7)	319 (87.2)	184 (80.7)	**0.045**
General	91 (15.3)	47 (12.8)	44 (19.3)	
ASA score	2.0 (2.0–3.0)	2.0 (2.0–3.0)	3.0 (2.0–3.0)	**<0.001**
Parker Mobility Score	6.0 (4.0–9.0)	6.0 (4.0–9.0)	6.0 (4.0–8.0)	0.018

Data are presented as median (interquartile range) or number (%). This table includes 594 patients with available data for the demographic and clinical variables shown. Because missingness differed across variables, its denominator differs from the laboratory comparison in Table 2. BMI and anesthesia type were assessed in unadjusted comparisons but were not included in the multivariable regression models. Higher Parker Mobility Scores indicate greater pre-fracture mobility.

**Table 2:** Laboratory Parameters According to Postoperative ICU Admission.

Variable	No ICU (*n* = 369)	ICU (*n* = 228)	*p*-Value
Admission Hb, g/dL	11.6 (10.4–12.9)	10.8 (9.6–12.1)	**<0.001**
Postoperative Hb (day 0), g/dL	10.1 (9.0–11.4)	9.7 (8.6–10.7)	**<0.001**
Postoperative Hb (day 1), g/dL	10.0 (9.2–10.8)	9.4 (8.6–10.2)	**<0.001**
Delta Hb (admission–postoperative day 1), g/dL	1.7 (0.6–2.5)	1.4 (0.4–2.5)	0.169
RDW-CV, %	14.7 (13.1–16.0)	16.6 (13.9–17.5)	**0.004**
CRP, mg/L	6.5 (2.2–29.9)	15.3 (3.6–62.2)	**<0.001**
WBC, ×10^3^/µL	11.0 (8.2–14.7)	10.4 (7.5–15.5)	0.645
Number of PRBC units transfused	1.0 (0.0–2.0)	2.0 (0.0–3.0)	**<0.001**

Data are presented as median (interquartile range). Laboratory comparisons were performed on 597 patients; one patient was excluded because of missing laboratory data. Because missingness differed across variables, this denominator differs from the clinical comparison in Table 1. Admission Hb, RDW-CV, CRP, and WBC values were obtained at initial hospital presentation. Hb: hemoglobin; RDW-CV: red blood cell distribution width-coefficient of variation; CRP: C-reactive protein; WBC: white blood cell count; PRBC: packed red blood cell.

**Table 3:** Characteristics According to 1-Month Mortality.

Variable	Survivors (*n* = 555)	Non-Survivors (*n* = 41)	*p*-Value
Age, years	84.0 (76.0–90.0)	89.0 (84.0–94.0)	**<0.001**
Sex, *n* (%)			
Female	396 (71.4)	20 (48.8)	**0.004**
Male	159 (28.6)	21 (51.2)	
Surgical type, *n* (%)			
PFN	333 (60.0)	12 (29.3)	**<0.001**
Hemiarthroplasty	222 (40.0)	29 (70.7)	
ASA score	2.0 (2.0–3.0)	3.0 (2.0–3.0)	**0.044**
Parker Mobility Score	6.0 (4.0–9.0)	5.0 (4.0–7.0)	0.269
Admission Hb, g/dL	11.2 (10.1–12.6)	10.7 (9.5–12.7)	0.391
RDW-CV, %	15.3 (13.3–16.8)	15.4 (13.7–17.2)	0.756
CRP, mg/L	8.2 (2.6–38.1)	31.9 (3.7–83.6)	**0.004**
WBC, ×10^3^/µL	10.8 (7.7–14.8)	11.7 (8.5–18.5)	0.251
Number of PRBC units transfused	1.0 (0.0–2.0)	2.0 (1.0–3.0)	**0.003**

Data are presented as median (interquartile range) or number (%). One-month mortality status was reliably confirmed for 596 patients, who formed the mortality analysis cohort. Higher Parker Mobility Scores indicate greater pre-fracture mobility.

**Table 4:** Adjusted Associations with 1-Month Mortality.

Variable	OR (95% CI)	*p*-Value
Age, per year	1.07 (1.02–1.12)	**0.005**
ASA score, per 1-point increase	1.40 (0.76–2.56)	0.281
Male sex (vs. female)	3.30 (1.66–6.56)	**<0.001**
Hemiarthroplasty (vs. PFN)	3.69 (1.78–7.64)	**<0.001**
Admission Hb, per 1 g/dL	1.09 (0.89–1.33)	0.422
RDW-CV, per 1%	0.99 (0.98–1.00)	0.155
CRP, per 10 mg/L	1.05 (1.01–1.10)	**0.030**
Number of PRBC units transfused, per unit	1.37 (1.11–1.70)	**0.004**

Model *n* = 596 (41 deaths). The model included age, ASA score, sex, surgical type, admission hemoglobin level, RDW-CV, CRP level, and number of PRBC units transfused. ORs for continuous predictors represent the unit increments specified in the table. Because the mortality analysis contained only 41 events, estimates from this model should be interpreted with caution. OR: odds ratio; CI: confidence interval; PFN: proximal femoral nailing; PRBC: packed red blood cell. Hemiarthroplasty versus PFN represents the combined fracture-treatment pathway because fracture type and procedure were perfectly collinear.

**Table 5:** Adjusted Associations with Postoperative ICU Admission.

Variable	OR (95% CI)	*p*-Value
Age, per year	1.04 (1.01–1.06)	**<0.001**
ASA score, per 1-point increase	2.04 (1.45–2.87)	**<0.001**
Male sex (vs. female)	1.47 (0.98–2.21)	0.064
Hemiarthroplasty (vs. PFN)	1.20 (0.83–1.74)	0.330
Admission Hb, per 1 g/dL	1.03 (0.91–1.16)	0.645
RDW-CV, per 1%	0.998 (0.985–1.012)	0.808
CRP, per 10 mg/L	1.02 (0.99–1.05)	0.496
Number of PRBC units transfused, per unit	1.60 (1.36–1.88)	**<0.001**

Model *n* = 597 (228 postoperative ICU admissions). The model included age, ASA score, sex, surgical type, admission hemoglobin level, RDW-CV, CRP level, and number of PRBC units transfused. ORs for continuous predictors represent the unit increments specified in the table. BMI and anesthesia type were not entered into the adjusted model; adjusted ORs for these variables are therefore not available. In patients admitted to the ICU, all PRBC transfusions included in this model occurred before ICU admission. OR: odds ratio; CI: confidence interval; PFN: proximal femoral nailing; PRBC: packed red blood cell.

**Table 6:** Firth Penalized Logistic Regression Sensitivity Analysis for 1-Month Mortality.

Variable	Core Firth OR (95% CI)	*p*-Value	Parker-Adjusted Firth OR (95% CI)	*p*-Value
Age, per year	1.07 (1.02–1.12)	**0.004**	1.07 (1.02–1.12)	**0.006**
ASA score, per 1-point increase	1.41 (0.78–2.54)	0.259	1.41 (0.78–2.53)	0.257
Male sex (vs. female)	3.05 (1.56–5.96)	**0.001**	3.04 (1.56–5.93)	**0.001**
Hemiarthroplasty (vs. PFN)	3.33 (1.65–6.71)	**<0.001**	3.29 (1.64–6.62)	**<0.001**
Admission Hb, per 1 g/dL	1.09 (0.89–1.32)	0.421	1.09 (0.89–1.33)	0.419
RDW-CV, per 1%	0.99 (0.98–1.00)	0.208	0.99 (0.98–1.00)	0.209
CRP, per 10 mg/L	1.04 (1.01–1.07)	**0.016**	1.04 (1.01–1.07)	**0.016**
Number of PRBC units transfused, per unit	1.36 (1.11–1.68)	**0.004**	1.36 (1.10–1.68)	**0.004**
Parker Mobility Score, per 1-point increase	—	—	1.00 (0.87–1.14)	0.956

The core Firth model included the same eight covariates as the conventional mortality model. The Parker-adjusted Firth model additionally included the pre-fracture Parker Mobility Score. Both Firth models used *n* = 596 patients (41 deaths); Parker Mobility Score was available for all 598 patients and therefore did not reduce the mortality analysis sample. Fracture category was not entered separately because it was perfectly collinear with surgical procedure in this cohort. OR: odds ratio; CI: confidence interval; PFN: proximal femoral nailing; PRBC: packed red blood cell.

## Data Availability

The datasets generated and/or analyzed during the current study are available from the corresponding author on reasonable request, subject to applicable institutional and ethical requirements.
